# School attendance and sexual and reproductive health outcomes among adolescent girls in Kenya: a cross-sectional analysis

**DOI:** 10.1186/s12978-023-01577-0

**Published:** 2023-02-06

**Authors:** Sai Surabi Thirugnanasampanthar, Lonnie Embleton, Erica Di Ruggiero, Paula Braitstein, Clement Oduor, Yohannes Dibaba Wado

**Affiliations:** 1 Dalla Lana School of Public Health, Centre for Global Health, University of Toronto, 155 College Street, Toronto, ON M5T 3M7 Canada; 2 College of Health Sciences, School of Public Health, Division of Epidemiology, Moi University, Eldoret, Kenya; 3 Academic Model Providing Access to Healthcare, Eldoret, Kenya; 4 African Population and Health Research Center, P. O. Box 10787-00100, Nairobi, Kenya; 5 Present Address: Department of Global Health and Health System Design, Icahn School of Medicine at Mount Sinai, Arnhold Institute for Global Health, New York, USA

**Keywords:** Sexual and reproductive health, Adolescent girls, Adolescent pregnancy, Contraceptives, School attendance, Kenya, Comprehensive sexuality education

## Abstract

**Background:**

Given the high burden of adverse sexual and reproductive health outcomes (SRH) and low levels of school attendance among adolescent girls in Kenya, this study sought to elucidate the association between school attendance and SRH outcomes among adolescent girls in Homa Bay and Narok counties.

**Methods:**

This study uses baseline quantitative data from the mixed-methods evaluation of the In Their Hands (ITH) program which occurred between September to October 2018 in Homa Bay and Narok counties. In total, 1840 adolescent girls aged 15–19 years participated in the baseline survey, of which 1810 were included in the present analysis. Multivariable logistic regression models were used to assess the association between school attendance (in- versus out-of-school) and ever having sex, condom use during last sex, and ever pregnant, controlling for age, orphan status, income generation, religion, county, relationship status, and correct SRH knowledge.

**Results:**

Across the 1810 participants included in our study, 61.3% were in-school and 38.7% were out-of-school. Compared to adolescent girls who were in-school, those out-of-school were more likely (AOR 5.74 95% CI 3.94, 8.46) to report ever having sex, less likely (AOR: 0.21, 95% CI 0.16, 0.31) to have used a condom during their last sexual intercourse, and more likely (AOR: 6.98, 95% CI 5.04, 9.74) to have ever been pregnant.

**Conclusions:**

School attendance plays an integral role in adolescent girls’ SRH outcomes, and it is imperative that policy actors coordinate with the government and community to develop and implement initiatives that support adolescent girls’ school attendance and education.

## Plain Language Summary

Adolescent girls in Kenya experience poor sexual and reproductive health outcomes (SRH) and have low levels of educational attainment. In this study, we aimed to examine if school attendance was associated with sexual and reproductive health outcomes among adolescent girls aged 15 to 19 years in Homa Bay and Narok counties in Kenya. The study used data from the evaluation of the In Their Hands (ITH) program which occurred between September to October 2018. A total of 1840 adolescent girls years participated in the baseline survey, of which 1810 were included in this study. We found that compared to adolescent girls in-school, those who were out-of-school were significantly more likely to report ever having sex, less likely to have used a condom during their last intercourse, and more likely to have ever been pregnant. This study found that attending school plays an important protective role in the SRH of adolescent girls. Therefore, it is important to develop and implement initiatives to support adolescent girls’ school attendance, and ultimately their education attainment.

## Introduction

Adolescence occurring from 10 to 19 years of age, is a developmentally critical period, and is characterized by rapid physical, cognitive, and psychosocial growth, including the process of puberty, which comes with adolescent-specific sexual and reproductive health (SRH) needs. During this influential period, adolescents’ health and well-being is highly shaped by the structural, social, and cultural contexts of where they live [1, 2]. Adolescent girls face a myriad of structural and social inequities that make them vulnerable to poor SRH outcomes, including poverty, gender inequities, differential power dynamics in relationships, stigma, and a lack of educational and economic opportunities [3, 4].

Education is a core structural determinant of health [5]. Educational attainment (e.g., completion of educational milestones such as primary or secondary school or higher education diplomas and degrees) has impacts across the life course and influences girls’ empowerment, choice of occupation and impacts opportunities for employment and personal income over time [5–7]. Completing secondary school and pursuing higher education works to reduce socioeconomic and health inequities that stem from poverty by leading to stable jobs, higher incomes, and wealth accumulation, while also providing long-term sources of social support, improving access to healthcare, and influencing health practices [6, 8, 9]. There is an abundance of evidence that demonstrates the association between lower educational attainment and poor health outcomes [6, 9, 10], including among adolescents [11]. However, the schooling process itself and not merely educational attainment has been under studied, but likely plays a significant role in the relationship between education and health outcomes [6]. Few studies have explicitly focused on exploring the association between school attendance (e.g., being in versus out-of-school) and SRH outcomes among adolescent girls. This is an important gap, as educational outcomes are often gendered, with fewer girls in Kenya enrolled in secondary school than boys, thereby differentially influencing health outcomes across genders [12]. The 2014 Kenya Demographic and Household Survey (KDHS) found a lower proportion of adolescent girls (68%) compared to adolescent boys (78%) who had attended school at any one time during the year [13]. In addition, adolescent girls had a lower completion rate of upper secondary schooling in comparison to adolescent boys, with rates at 39% and 46% respectively [14].

Gender inequities are also abundant in relation to adolescent SRH. In comparison to boys, adolescent girls in Kenya are more likely to be married before the age of 18, have a higher prevalence of sexually transmitted infections (STIs), and disproportionately bear the burden of new adolescent HIV infections [8]. There is a substantial unmet need for contraception among adolescent girls in the country, and adolescent girls account for 14% of all childbirths in Kenya, with two-thirds of adolescent pregnancies being undesired [15]. Generally, adolescent girls who experience early marriage and those who have commenced childbearing, leave school and are unable to return to complete their education [16, 17]. Remaining enrolled in and attending school is likely a critical protective factor in adolescent girls’ SRH, and long-term health and well-being.

Attending school offers opportunities for social support and comprehensive sexuality education (CSE), while contributing to empowering girls and young women [18, 19]. At the same time, attending school may reduce the likelihood of adolescent girls entering age-disparate relationships, delay their sexual debut and first pregnancy, and protect them from acquiring STIs [20]. Data from several sub-Saharan African (SSA) countries, found that out-of-school adolescents were at an increased risk of poor SRH outcomes and low healthcare utilization. Compared to those out-of-school, in-school adolescents were 51% less likely to have had sexual intercourse and 21% more likely to use a condom at last sex [20]. Additionally, a systematic review and meta-analysis found that adolescent girls who were out-of-school were two times more likely to start childbearing than those who were in school [21].

While school may be protective in most situations, in some cases the school environment itself may contribute to adverse SRH outcomes. For instance, teachers may perpetrate sexual violence, be ill prepared to deliver accurate and quality CSE, while schools may also lack acceptable sanitation and hygiene resources, such as menstrual pads, for girls and young women experiencing menstruation [22, 23]. Further, while primary school is free, there are other costs, such as exam fees, uniforms, and lunch costs. At times, these additional fees result in adolescent girls and young women engaging in transactional sex for material and/or monetary support, leaving them vulnerable to poor SRH outcomes [22]. Thus, the relationship between school attendance and SRH outcomes is complex and multi-faceted.

The COVID-19 pandemic has further exacerbated adolescent girls’ barriers to schooling and SRH outcomes as a result of pandemic-related school closures. Adolescent girls in western Kenya who experienced school disruptions due to COVID-19 were more likely to be sexually active, and experienced three times the risk of dropping out, and twice the risk of becoming pregnant before secondary school completion than adolescent girls before COVID-19 [24]. This evidence suggests that attending and staying in school plays a strong protective role in adolescent girls’ SRH in this context.

There is a clear need to better characterize the role of *school attendance* on adolescent girls’ SRH outcomes in Kenya. Educational attainment (i.e., the highest level of education an individual has completed) is often conceptualized as a confounder and adjusted for in analyses. Studies have seldomly examined how school attendance, as the main exposure of interest, is associated with SRH outcomes. It is imperative to understand how school attendance impacts SRH outcomes in adolescent girls to provide further evidence for policies and programs that support keeping adolescent girls in school, the development of comprehensive sexuality education (CSE), and designing school-based interventions to address poor SRH outcomes among adolescent girls. Given the high burden of poor SRH outcomes in Kenya and lower levels of educational attainment among adolescent girls, this study sought to examine the association between school attendance and SRH outcomes among adolescent girls in Homa Bay and Narok counties in Kenya. We hypothesized that adolescent girls’ currently attending school would be less likely to have ever had sex and ever be pregnant, and more likely to use a condom at last sex.

## Materials and methods

### Study design

The present cross-sectional analysis uses baseline data from the In their Hands (ITH) evaluation to examine the association between school attendance and SRH outcomes. The ITH program was implemented in Kenya between April 2017 to March 2020 across eighteen counties to increase adolescent’s access to and use of high-quality SRH services through a targeted intervention. The evaluation utilized a mixed-method cross sectional design that collected both quantitative and qualitative baseline data concurrently in the periods between September to October 2018.

### Study setting

Homa Bay and Narok counties were selected for the evaluation as the ITH intervention had not yet been implemented at baseline, and the counties had the highest prevalence of adolescent pregnancy of the 18 counties where the program was to be implemented. Homa Bay has a population of 1,131,950, and adolescents aged 10–19 comprise 28% of the population. Similarly, adolescents make up 26% of the total Narok population (1,157,873) [25].

### Study population and sampling approach

Adolescent girls aged 15 to 19 years were recruited to participate in the intervention. The inclusion criteria were being an adolescent girl aged between 15 and 19 years, a usual resident in the study area (has lived at least 6 months preceding the study), and being a member of a sampled household. On the other hand, students who are in boarding schools and mostly stay away from their parents, and adolescents who were not competent for informed consenting were excluded from the study.

The sampling approach included purposive selection of Homa Bay and Narok counties. Three sub-counties were selected within each of these two counties. Within each of the sub-counties, three wards were selected based on the distribution of ITH affiliated heath facilities. For each of the health facilities that were sampled, catchment villages served by the facility was identified. Resultantly, 22 and 24 villages were sampled in Narok and Homa Bay counties, respectively. Household listings of each of the villages were used to identify households with adolescent girls. Through random selection, only one adolescent girl was interviewed from each household where at least an adolescent girl was listed.

The baseline dataset included 1840 adolescent girls aged 15–19 years who were residents in the study areas for at least 6 months prior to the study. The response rate for participation was 97%. The survey targeted 1897 adolescents. 1840 adolescent girls were successfully recruited and participated in the cross-sectional survey; 57 (3%) adolescent girls selected did not participate due to lack of parental consent, unavailability, or refusal to participate. The present analysis was restricted to adolescent girls who had ever attended school. Participants who had never attended school (n = 30) were excluded. Resultantly, a total of 1810 participants were included in this analysis.

### Ethical considerations

The study protocol and data collection instruments were reviewed and approved by AMREF Health Africa Ethics and Scientific Review Committee. Furthermore, research clearance was granted by Kenya’s National Commission for Science, Technology and Innovation. Additional approvals were obtained from local commissioners and the Ministries of Health and Education in the respective counties where the study was conducted in. Individual consent was sought from adolescents who were aged 18–19 years or were emancipated minors. For adolescents younger than 18 years old, both parental/guardian consent and adolescent assent was obtained prior to interviews and data collection.

### Quantitative data collection

Quantitative data was collected from a representative sample of adolescent girls living in urban and rural ITH program areas. Research assistants were trained on all aspects of the study protocol. An interviewer-administered structured questionnaire was used to collect quantitative data to understand adolescent girls’ use of SRH outcomes and services, as well as their access to information, prior to the implementation of the ITH program. Interviewers used a tablet to collect the information during the face-to-face interviews. These interviews were conducted in a private setting to ensure confidentiality. While the study tools were programmed in the SurveyCTO in both English and Kiswahili, respondents who consented to participate were asked about their preferred language of interview. Furthermore, since the interviewers were recruited on their knowledge of the study area and ability to communicate in any of the dominant local languages spoken in any of the study area, where the respondents were not fluent in both languages of interview, such interviews were conducted in the local languages by competent interviewers. The questionnaire was piloted to assess consistency, appropriateness, readability, and ease of understanding of the questions.

### Variables

The main exposure of interest was current school attendance, defined as either currently attending school or not, at the time of the survey. This was a self-reported measure assessed at baseline by asking participants ‘*Are you currently attending school?*’ and was reported as a binary variable: currently in-school and out-of-school. Our SRH outcomes of interest were: (1) ever had sexual intercourse, (2) condom use during last sexual intercourse, and (3) ever been pregnant. Responses for these three main outcomes were reported and categorized as binary categorical variables (yes/no). Of those who reported ever having sexual intercourse, participants were asked if a condom was used during last sexual intercourse and if they have ever been pregnant.

To guide the analysis, a conceptual model was developed a priori through the identification of potential confounders and effect measure modifiers (EMM). Based on the current literature, the present study aimed to adjust for predictors of SRH outcomes and sociodemographic variables: including age, orphan status, engagement in income generating activities, religion, county, and relationship status. In addition, we hypothesized that having correct SRH knowledge may be an EMM, as knowledge is necessary but not sufficient to improve adolescent’s health promoting behavior [26].

Age was self-reported and later recoded as a binary categorical variable [15–19]. Participants’ county was documented by interviewers. To determine orphan status, participants were asked if their biological parents were alive, and orphan status was categorized into single, double, or non-orphaned. A single orphan was defined as a child whose mother (maternal), or father (paternal) had died or was absent from their life. A double orphan was defined as a child having both parents who had died or were absent from their life. If participants reported having both parents alive and/or present in their life, they were categorized as not orphaned. Participants were asked if they had engaged in any activities for which they got money or any kind of payment in the last 6 months (yes/no) to measure engagement in income generation. Participants were asked what religion they practiced and categorized as Catholic, Protestant/Other Christian, Islam/tradition/no religion. Relationship status was classified into the following categories: currently married/in union, has boyfriend/engaged, never been in a relationship, currently not/had past boyfriend, divorced/separated/widowed. SRH knowledge was assessed by asking participants which time a woman is more likely to become pregnant if she has sexual relations. Participants who answered ‘2 weeks after her period’ were classified as having correct menstrual cycle knowledge. All other options that were reported were classified as incorrect. As a result, correct menstrual cycle knowledge was recoded into a binary variable (correct/incorrect).

### Statistical analysis

Descriptive statistics were used to determine frequencies and proportions for categorical variables and means and standard deviations (SDs) for continuous variables. We conducted bivariate logistic regression to examine the relationship between school attendance (in-school vs. out-of-school), SRH outcomes (ever had sexual intercourse, condom use during last sexual intercourse, and ever been pregnant), and potential confounders including, age, county, orphan status, religion, relationship status, income generation, and correct menstrual cycle knowledge. We then conducted multiple logistic regression producing three models, one for each SRH outcome of interest, controlling for confounders, to produce adjusted odds ratios (AOR) and 95% confidence intervals (CIs). Multicollinearity of the models was assessed at a threshold of 0.8, and not observed. EMM was assessed for correct SRH knowledge. Likelihood ratio tests were performed with full and nested models to determine whether interaction terms should be included; if they were not statistically significant at an alpha level of 0.05, then the reduced model was chosen as in this case. However, given that correct SRH was statistically significant, it was included in all three models as a covariate.

To ensure data quality, all variables were assessed for missingness. If variables had a low proportion of missingness (characterized as less than 5%), observations were assumed to be missing completely at random [27]. As no variables had greater than 5% of missing data, a complete case analysis was employed as it was likely to not bias the estimate and sample size of each model run. All analyses were conducted using RStudio Version 1.4.1106.

## Results

### Sociodemographics

Resultantly, this analysis included a total of 1810 adolescent girls, of whom 61% were currently in-school and 39% were out-of-school (Table 1). There was a greater proportion of adolescent girls aged 18–19 years who were out-of-school (80%), compared to those aged 15–17 years (20%). Over half (56%) of the participants reported that primary school was the highest level of attended, with 41% reporting secondary school and 2% higher education. The primary reasons given for no longer attending school included the inability to pay school fees (43%), having been pregnant/had a baby (34%), and having gotten married (22%). A small proportion of respondents (7%) were out-of-school due to secondary completion, and reported they were awaiting entry to post-secondary education. A higher proportion of out-of-school adolescent girls (37%) reported having engaged in income generating activities in the past 6 months in comparison to those in-school (10%), although most study participants (80%), did not engage in income generating activities. Half (49%) of out-of-school participants reported being married/in a union. In contrast, 45% of participants currently in-school reported never having been in a relationship.

**Table 1:** Participants’ sociodemographic characteristics stratified by current school attendance

Sociodemographics	School attendance		
	Yes (n = 1110) n (%)	No (n = 700) n (%)	Total (N = 1810) N (%)
Age			
15–17	833 (75.0)	141 (20.1)	974 (53.8)
18–19	277 (25.0)	559 (79.9)	836 (46.2)
County			
Narok	414 (37.3)	338 (48.3)	752 (41.5)
Homa Bay	696 (62.7)	362 (51.7)	1058 (58.5)
Highest level of school attended			
Primary	665 (59.9)	356 (50.9)	1021 (56.4)
Secondary	416 (40.0)	333 (48.7)	749 (41.4)
Vocational/college/university	28 (2.5)	8 (1.1)	36 (2.0)
Missing	1 (0.1)	3 (0.4)	4 (0.22)
Religion			
Catholic	248 (22.3)	138 (19.7)	386 (21.3)
Protestant/christian	839 (75.6)	551 (78.7)	1390 (76.8)
Islam/tradition/no religion	23 (2.1)	11 (1.6)	34 (1.9)
Orphan status			
Both parents alive	799 (72.0)	401 (57.3)	1200 (66.4)
Both parents not alive	53 (4.8)	96 (13.7)	149 (8.2)
Maternal orphan	46 (4.1)	34 (4.9)	80 (4.5)
Paternal orphan	212 (19.1)	169 (24.1)	381 (21.0)
Income generation past 6 months			
Yes	111 (10.0)	259 (37.0)	370 (20.4)
No	999 (90.0)	441 (63.0)	1440 (79.6)
Relationship status			
Currently married/in union	17 (1.5)	345 (49.3)	362 (20.0)
Has boyfriend/engaged	439 (39.6)	236 (33.7)	675 (37.3)
Never been in a relationship	500 (45.0)	35 (5.0)	535 (29.6)
Currently not/had past boyfriend	147 (13.2)	72 (10.3)	219 (12.1)
Divorced/separated/widowed	6 (0.5)	12 (1.7)	18 (1.0)
Missing	1 (0.1)	0 (0.0)	1 (0.1)

### SRH knowledge and sources of information

Table 2 summarizes sources of SRH information and knowledge for study participants. Participants, both in-school and out-of-school, reported primarily relying on teachers (76% vs. 46%), their mother (26% vs. 21%) and friends (27% vs. 35%) for SRH information and knowledge. Overall, over half of all participants had received information about SRH services in the past year, and information about pregnancy and HIV in the past few months. In general, participants were knowledgeable about the most effective method to prevent STIs and pregnancy. However, a large proportion of both in-school (75%) and out-of-school participants (68%), responded that they think that some family planning methods cause infertility. Most participants (87%) were aware that there are days in the menstrual cycle when a woman is more likely to get pregnant. However, only a few participants (14%) were able to report the correct days during the menstrual cycle that a woman is more likely to get pregnant, with a higher proportion of those out-of-school answering correctly (18%).

**Table 2:** Sexual and reproductive health knowledge and information sources among adolescent girls in Kenya stratified by school attendance

SRH sources of information and knowledge	School attendance		
	Yes (n = 1110) n (%)	No (n = 700) n (%)	Total (N = 1810) N (%)
Most important sources of SRH information			
School teacher	871 (78.5)	326 (46.6)	1197 (66.1)
Mother	284 (25.6)	146 (20.9)	430 (23.8)
Father	16 (1.4)	6 (0.9)	22 (1.2)
Siblings	91 (8.2)	47 (6.7)	138 (7.6)
Other family members	124 (11.2)	100 (14.3)	224 (12.4)
Friends	297 (26.8)	244 (34.9)	541 (29.9)
Doctors/healthcare providers	53 (4.8)	142 (19.9)	195 (10.6)
Radio/film/videos/television	62 (5.2)	75 (10.3)	137 (7.2)
Books/magazine	105 (9.5)	48 (6.9)	153 (8.5)
Other	91 (8.6)	66 (10.3)	157 (9.2)
Received information about SRH services in the past year			
Yes	590 (53.2)	365 (52.1)	955 (52.8)
No	520 (46.8)	335 (47.9)	855 (47.2)
Received information about pregnancy in the past few months			
Yes	673 (60.6)	398 (56.9)	1071 (59.2)
No	437 (39.4)	302 (43.1)	739 (40.8)
Received information about HIV in the past few months			
Yes	813 (73.2)	466 (66.6)	1279 (70.7)
No	297 (26.8)	234 (33.4)	531 (29.3)
Most effective family planning method to prevent STIs			
IUD/implant/injectables	68 (6.1)	46 (6.6)	114 (6.3)
Male/female condom	840 (75.7)	590 (84.3)	1430 (79.0)
Daily pill	31 (2.8)	12 (1.7)	43 (2.4)
Emergency contraception	12 (1.1)	2 (0.3)	14 (0.8)
Natural methods^+^	20 (1.8)	4 (0.6)	24 (1.3)
Abstinence	32 (2.9)	3 (0.4)	35 (1.9)
Don’t know/nothing	107 (9.6)	43 (6.1)	150 (8.3)
Most effective family planning method to prevent pregnancy			
IUD/implant/injectables	460 (41.4)	462 (66.0)	922 (50.9)
Male/female condom	335 (30.2)	135 (19.3)	470 (26.0)
Daily pill	79 (7.1)	41 (5.9)	120 (6.6)
Emergency contraception	17 (1.5)	11 (1.6)	28 (1.5)
Natural methods^+^	59 (5.3)	17 (2.4)	76 (4.2)
Abstinence	38 (3.4)	4 (0.6)	42 (2.3)
Don’t know	122 (11.0)	30 (4.3)	152 (8.4)
Some family planning methods cause infertility			
Disagree	282 (25.4)	224 (32.0)	506 (28.0)
Agree/don’t know	828 (74.6)	476 (68.0)	1304 (72.0)
There are days in the menstrual cycle when a woman is more likely to become pregnant if she has sex			
Yes	920 (82.9)	650 (92.9)	1570 (86.7)
No	158 (14.2)	43 (6.1)	201 (11.1)
Missing/no response	32 (2.9)	7 (1.0)	39 (2.2)
Time in menstrual cycle a woman is more likely to get pregnant			
	*n* = *920*	*n* = *650*	*N* = *1570*
Two weeks after her period	108 (11.7)	118 (18.1)	226 (14.4)
Other times/don’t know	812 (88.3)	532 (81.8)	1344 (85.6)

*IUD* Intrauterine device; *Natural* *methods* standard days/safe days and withdrawal; *Other times* before her period begins, during her period, right after her period has ended, other

### Sexual activity and reproductive health outcomes

Among all study participants, 60% reported ever having sexual intercourse, of whom 59% were out-of-school (Table 3). Of those who reported ever having sexual intercourse, the mean (SD) age at sexual debut was 14.80 (1.70) and 15.20 (1.75) years old for those in-school and out-of-school, respectively. Participants who were out-of-school had a higher mean (SD) number of lifetime sexual partners (2.13 ± 3.97) compared to those in school (1.52 ± 0.82). Among study participants who have ever had sex, most (82%) reported being sexually active in the past 12 months, with fewer in-school versus out-of-school (73% vs. 88%) participants reporting being sexually active. Less than half (47%) of participants reported condom use at last sexual encounter, with fewer out-of-school girls reporting this than in-school girls (32% vs. 69%) Around half all participants (51%) reported that they were doing something or reported using a contraceptive method to prevent pregnancy. Among those currently using contraceptives, the most common contraceptives used were condoms (54%), IUD/injectables/impact (23%) and emergency contraception (19%). Almost three quarters (74%) of out-of-school participants reported ever being pregnant, in comparison to approximately a quarter (24%) of those in-school. Most adolescent girls reported that their pregnancy was undesired at the time (78%). This differed by school status, with 96% of in-school adolescents reporting they wanted to wait or did not want the pregnancy. Across the participants that had reported ever being pregnant, there was a high proportion of participants who have given birth (85%).

**Table 3:** Sexual and reproductive health outcomes of adolescent girls in Kenya stratified by school attendance

SRH outcomes	School Attendance		
	Yes (n = 1110) n (%)	No (n = 700) n (%)	Total (N = 1810) N (%)
Ever had sexual intercourse			
Yes	444 (40.0)	636 (90.9)	1080 (59.7)
No	666 (60.0)	64 (9.1)	730 (40.3)
Age at sexual debut (mean ± SD)			
	*n* = *444*	*n* = *636*	*N* = *1080*
	14.80 ± 1.70	15.20 ± 1.75	15.00 ± 1.77
Lifetime number of sexual partners (mean ± SD)			
	*n* = *444*	*n* = *636*	*N* = *1080*
	1.52 ± 0.82	2.13 ± 3.97	1.88 ± 3.08
Sexually active in the past 12 months			
Yes	326 (73.4)	561 (88.2)	887 (82.1)
No	118 (26.6)	75 (11.8)	193 (17.9)
Condom use during last sexual intercourse			
Yes	305 (68.7)	203 (31.9)	508 (47.0)
No	139 (31.3)	433 (68.1)	572 (53.0)
Currently doing something or using a contraceptive method to prevent pregnancy			
Yes	237 (53.4)	309 (48.6)	546 (50.6)
No	197 (44.4)	324 (50.9)	521 (48.2)
Missing/no response	10 (2.3)	3 (0.5)	13 (1.2)
Contraceptive method currently using			
	*n* = *237*	*n* = *309*	*N* = *546*
IUD/injectables/implant	24 (10.1)	102 (33.0)	126 (23.1)
Male/female condom	196 (82.7)	101 (32.7)	297 (54.4)
Daily pill	1 (0.4)	2 (0.6)	3 (0.5)
Emergency contraception	10 (0.9)	94 (30.4)	104 (19.0)
Natural methods^+^	6 (2.5)	10 (3.2)	16 (2.9)
Ever pregnant			
	*n* = *444*	*n* = *636*	*N* = *1080*
Yes/currently pregnant	107 (24.1)	468 (73.6)	575 (53.2)
No	337 (75.9)	168 (26.4)	505 (46.8)
Pregnancy desire			
	*n* = *107*	*n* = *468*	*n* = *575*
Wanted	4 (3.7)	116 (24.8)	120 (20.9)
Wanted to wait/wanted no more	103 (96.3)	346 (73.9)	449 (78.1)
Didn’t mind either way	0 (0.0)	6 (1.3)	6 (1.0)
Ever given birth			
	*n* = *107*	*n* = *468*	*n* = *575*
Yes	83 (77.6)	404 (86.3)	487 (84.7)
No	23 (21.5)	64 (13.7)	87 (15.1)
Missing/no response	1 (0.9)	0 (0.0)	1 (0.2)

*IUD* intrauterine device; *Natural methods* standard days/safe days and withdrawal

Table 4 presents unadjusted and adjusted analyses for the three SRH outcomes, ever sex, condom use at least sex, and ever pregnant among in-school and out-of-school adolescent girls. Compared to adolescent girls who were in-school, those out-of-school had 5.74 times higher odds (95% CI 3.94, 8.46) of ever having sex, adjusting for age, county, religion, orphan status, income source, relationship status, and correct menstrual cycle knowledge. Furthermore, compared to adolescent girls who were in-school, those out-of-school were less likely (AOR: 0.22, 95% CI 0.16, 0.31) to have used a condom during their last sexual intercourse and more likely (AOR: 6.98, 95% CI 5.04, 9.74) to have ever been pregnant, while adjusting for potential confounders.

**Table 4:** Unadjusted and adjusted multivariable logistic regression models examining the association with current school attendance and sexual and reproductive health outcomes among adolescent girls in Kenya

Covariates	Ever sex (N = 1539)		Condom use at last intercourse (N = 958)		Ever pregnant (N = 958)	
	Unadjusted OR (95% CI)	Adjusted AOR^a^ (95% CI)	Unadjusted OR (95% CI)	Adjusted AOR^a^ (95% CI)	Unadjusted OR (95% CI)	Adjusted AOR^a^ (95% CI)
School attendance						
Yes	*ref*	*ref*	*ref*	*ref*	*ref*	*ref*
No	14.91 (11.30, 19.95)	5.74 (3.94, 8.46)	0.21 (0.16, 0.28)	0.22 (0.16, 0.31)	8.77 (6.65, 11.65)	6.98 (5.04. 9.74)
Age						
15–17	*ref*	*ref*	*ref*	*ref*	*ref*	*ref*
18–19	8.43 (6.75, 10.59)	2.91 (2.13, 3.98)	0.54 (0.42, 0.69)	1.16 (0.83, 1.62)	3.66 (2.82, 4.75)	2.06 (1.47, 2.88)
County						
Narok	*ref*	*ref*	*ref*	*ref*	*ref*	*ref*
Homa bay	1.19 (0.99, 1.44)	1.92 (1.42, 2.60)	3.40 (2.64, 4.40)	2.98 (2.20, 4.05)	0.76 (0.60, 0.97)	1.11 (0.81, 1.53)
Religion						
Catholic	*ref*	*ref*	*ref*	*ref*	*ref*	*ref*
Protestant/christian	1.16 (0.92, 1.45)	0.94 (0.66, 1.33)	0.83 (0.62, 1.12)	0.88 (0.63, 1.25)	1.67 (1.24, 2.25)	1.77 (1.22, 2.56)
Islam/traditional/no religion	1.05 (0.52, 2.19)	1.24 (0.43, 3.51)	1.00 (0.40, 2.53)	0.77 (0.25, 2.38)	1.57 (0.63, 4.04)	2.71 (0.83, 9.19)
Orphan Status						
Both parents alive	*ref*	*ref*	*ref*	*ref*	*ref*	*ref*
Both parents not alive	2.24 (1.54, 3.32)	0.95 (0.54, 1.70)	0.93 (0.62, 1.40)	1.11 (0.69, 1.79)	1.94 (1.28, 2.99)	1.51 (0.90, 2.59)
Maternal orphan	1.36 (0.86, 2.19)	0.95 (0.47, 1.94)	0.77 (0.42, 1.35)	0.77 (0.39, 1.51)	0.97 (0.55, 1.71)	0.99 (0.49, 2.00)
Paternal orphan	1.68 (1.32, 2.15)	1.03 (0.72, 1.47)	1.39 (1.05, 1.86)	1.42 (1.01, 2.00)	1.31 (0.98, 1.75)	1.30 (0.91, 1.87)
Income generation past 6 months						
Yes	*ref*	*ref*	*ref*	*ref*	*ref*	*ref*
No	0.34 (0.26, 0.44)	0.73 (0.49, 1.09)	1.75 (1.33, 2.30)	0.89 (0.64, 1.25)	0.60 (0.46, 0.78)	1.11 (0.79, 1.57)
Relationship status						
Married/boyfriend/engaged	*ref*	*ref*	*ref*	*ref*	*ref*	*ref*
Never been in a relationship/currently not/divorced/separated/widowed	0.07 (0.05, 0.08)	0.13 (0.10, 0.17)	1.57 (1.16, 2.13)	1.26 (0.87, 1.81)	0.50 (0.37, 0.68)	0.83 (0.57, 1.23)
Correct menstrual cycle knowledge						
incorrect	*ref*	*ref*	*ref*	*ref*	*ref*	*ref*
Correct	2.29 (1.65, 3.21)	1.53 (1.00, 2.37)	0.68 (0.49, 0.94)	0.65 (0.45, 0.95)	1.33 (0.96, 1.86)	1.16 (0.79, 1.71)

*OR* Odds Ratio; *AOR* Adjusted Odds Ratio

^a^Estimates are adjusted for age, county, religion, orphan status, employment/income source, relationship status, and correct menstrual cycle knowledge

## Discussion

This analysis examined the association between school attendance and SRH outcomes among adolescent girls aged 15–19 years in Homa Bay and Narok counties in Kenya. Our results highlight three critical issues. First, our findings are congruent with our hypothesis that school attendance independently protects adolescent girls from poor SRH outcomes. Second, both in-school and out-of-school adolescent girls demonstrated gaps in SRH knowledge. Third, the study found that over half of all participants had ever been pregnant and revealed that the majority of adolescent pregnancies are undesired regardless of school status.

This study showed that compared to adolescent girls in school, those who were out-of-school had significantly higher odds of ever having sex, ever being pregnant, and lower odds of having used a condom during last sexual intercourse. Our findings are consistent with the current evidence base, including meta-analyses and observational studies, regarding the protective role school attendance has on adolescent health, especially for SRH outcomes [20, 21, 28]. Recently, De Neve et al. analyzed cross-sectional survey data from seven SSA countries and found that in-school adolescents were less likely to report ever having sexual intercourse and more likely to have used a condom during their last sexual intercourse [20]. Furthermore, a systematic review and meta-analysis by Kassa et al., which examined the prevalence and determinants of adolescent pregnancy in Africa, also reported that adolescent girls who were not attending school were twice as likely to be pregnant than those in-school [21]. The present study adds to the literature of how school attendance itself and not only educational attainment is independently associated with adolescent SRH outcomes, potentially signifying the underlying beneficial role the schooling process has on adolescents [6].

A substantial number of adolescent girls in this study were not in-school, and many were still in primary level education despite being aged 15 and above, suggesting they experienced substantial disruptions or delays in their education. Participants in this study reported that the most common reasons for not attending school were not being able to pay school fees, having gotten married, and having become pregnant. Though Kenya had implemented a free primary education policy in 2003, it is evident that there are persistent social and structural barriers impacting school enrolment and attendance, such as poverty and gender norms, which continue to perpetuate educational disparities [29]. It is imperative to reduce structural and social barriers to both primary and secondary education for adolescent girls. A body of evidence suggests that conditional and unconditional cash transfers increase school enrolment and attendance [30]. Kenya’s cash-transfer to orphaned and vulnerable children (CT-OVC) has had important impacts on secondary school enrolment for young women and has protected against early pregnancy [31]. Expanding the reach of the CT-OVC program to provide universal coverage to disadvantaged families is a key strategy to increasing adolescent girls’ school enrolment, attendance, and educational attainment, while reducing early pregnancy and other disadvantageous SRH outcomes. Beyond the CT-OVC, removing financial barriers to schooling, such as tuition, exam fees, and uniform costs for primary and secondary students, has proven to reduce the dropout rate for vulnerable children and adolescents in western Kenya [32]. This further supports the need for policy to ensure primary and secondary education is free of charge to all children and adolescents in Kenya to remove structural barriers to education that also fuel long-term health and social impacts. Other targeted evidence-based approaches exist that may be adapted and implemented in the Kenyan context to ensure girls remain enrolled in and attending school. One example is the provision of scholarships that cover fees and daily tuition for secondary education, which had a significant impact on secondary school completion among young women in Ghana [33]. Further, scholarships in this study reduced pregnancies and unwanted pregnancies, and had a longer-term effect on delayed child-bearing and women’s access to jobs [33]. The evidence is strong that economic and social protection strategies work to support adolescent girls’ school enrolment, attendance, and educational attainment, and at the same time improve SRH outcomes for vulnerable girls and therefore should be adopted as national policy.

Finally, interventions need to be developed to support adolescent girls who have left school due to pregnancy, to return to school to complete their primary and secondary education. In the present analysis, approximately a third of the out-of-school girls left school due to pregnancy. Returning to school after childbearing for adolescent girls is uncommon, one study in western Kenya found that 91% of schoolgirls who became pregnant dropped out [34]. A recent study that aimed to assess the factors that are associated with adolescent mothers returning to school in South Africa revealed that lacking childcare presents a significant barrier for adolescent mothers’ return to school [35]. As such, policymakers should work to support the return and retention of adolescent mothers in school through multifaceted interventions, which include access to childcare services.

Teachers were cited as the most important source of SRH information for both in-school and out-of-school participants in this study. However, when asked specific knowledge based SRH questions, it showed that most participants had incorrect knowledge and were unaware or misinformed. A large proportion of participants believed that some family planning methods cause infertility and were also unaware of the correct time in a menstrual cycle a woman is more likely to get pregnant. The lack of comprehensive sexuality education (CSE) in Kenya is a cause for concern, as the knowledge and information gained can be used to not only protect adolescent girls from harmful SRH outcomes, but it has also been shown to improve self-confidence, positively change attitudes, and build self-efficacy [36, 37]. CSE adopts as right-based and gender-focused approach that equips adolescents with the knowledge, skills, and attitudes that will allow them to have a positive view of their SRH [36]. The United Nations Educational, Scientific and Cultural Organization (UNESCO) and the United Nations Population Fund had conducted a review of the sexual education curriculum in Kenya and reported that the curricula promoted abstinence, lacked adequate information regarding contraceptives, abortion, and sexual health services [36]. Important CSE topics such as gender-based violence and intimate partner violence are also often excluded. Additionally, students in Kenya have reported that CSE often only focuses on sexual and reproductive physiology and prevention of STIs, such as HIV [36]. Implementation of CSE in some SSA countries is met with resistance, as topics such as gender inequality, adolescent sexuality, and abortion, conflict with dominant gender norms, cultural and religious beliefs [38]. However, it is critical adolescent girls have accurate and unbiased information on SRH. Working to reduce stigma associated with adolescent sexuality in the community is an important component to improving CSE. It is also imperative that teachers have the appropriate information and resources to teach students about a breadth of SRH topics, as a recent report found that 68% of teachers in Kenya felt they needed more training [36]. Lastly, with an increase of out-of-school adolescent girls because of the COVID-19 pandemic, strategies to ensure that CSE is inclusive and accessible to out-of-school adolescent’s warrants further inquiry and action. In summary, our findings underscore how schools serve as an important policy lever that can deliver and provide information regarding SRH services and knowledge to adolescent girls, but that alternative avenues for delivering CSE are also needed to reach the out-of-school adolescent population.

Finally, our analysis found a very high proportion of adolescent girls had ever been pregnant, with over three quarters of adolescent girls, both in- and out-of-school, reporting that the pregnancy was undesired at the time. As previously explored by Ajayi et al. in this context, adolescent girls reported that poor knowledge of contraceptive methods, misinformation, and gender and relationship issues between young men and women were the primary reasons for unintended pregnancy [39]. There is a substantial unmet need for contraception among young women in Kenya, particularly among unmarried sexually active adolescent girls [15, 40]. As such, many adolescent girls either use no contraceptives or use natural planning methods that have low effectiveness, resulting in a high prevalence of undesired pregnancy among this group. Most commonly, contraception is used by adolescent girls and young women who have commenced childbearing, and therefore its use is no longer heavily stigmatized and adolescent and girls and young women are counseled regarding contraceptive use post-partum [41]. There is a critical need for strategies to make modern contraceptives readily accessible and available to all adolescents, regardless of marital or childbearing status, while improving access to and provision of CSE, and adolescent and youth-friendly services to help reduce the number of unintended adolescent pregnancies [15]. While access to contraceptives is important, it is critical to also recognize how upstream sociocultural factors stigmatize contraceptive use, thereby influencing adolescent girls’ decisions of using contraceptives. Unmarried and sexually active adolescent girls are perceived negatively, and contraceptive use is seen as unacceptable [42, 43]. In addition, there exists widespread misinformation, that is rooted in social and gender norms, regarding hormonal contraceptive use among unmarried adolescent girls, resultantly instilling fear among this group. For example, there is a widespread belief that unmarried adolescent girls who use modern contraceptives would have fertility issues later in life [42, 43]. As such, it is crucial to destigmatize contraceptive use among adolescents within the community in Kenya. The widespread misinformation regarding contraceptive use further emphasizes the need for CSE for adolescents as well as community-based programmes and resources to challenge misconceptions about contraceptives and improve beliefs regarding adolescent sexuality.

Though our study found a strong association between school attendance and SRH outcomes, there are structural and social determinants of health, such as poverty (e.g., not being able to pay fees) and gender disparities that generate stark health disparities and influence both school attendance and SRH outcomes. As such, addressing poor SRH among adolescent girls requires multifaceted approaches. Ensuring girls enroll and attend school and complete their education is one of the many, but fundamental, avenues to improve health outcomes of adolescents, while concurrently working towards addressing other upstream structural and social factors (e.g., gender norms, stigma, and poverty) that also shape adolescent SRH.

## Study strengths and limitations

This study had several limitations. Firstly, data were self-reported and subject to social desirability bias, and it is likely study participants underreported their sexual practices. This is the case for unmarried adolescent girls who may have been hesitant to disclose sexual activity for fear of disapproval from community members. Additionally, the data may potentially suffer from both recall bias as well. Some information, such as age of the respondent may have been affected by reporting and recall bias. Next, a proportion (7%) of participants reported having completed secondary school and were awaiting to continue post-secondary education. These adolescent girls were categorized as ‘out-of-school’ for the purposes of this analysis; however, as they did not leave school due to drop-out, they may be characteristically similar to those in-school and may have minimally attenuated the association between current school attendance and SRH outcomes. Similarly, a proportion (34%) of adolescent girls who were out-of-school reported that they left school due to pregnancy, highlighting that pregnancy while in-school contributed to their drop-out, thereby contributing to the association between being out-of-school and ever pregnant. Finally, the data utilized in this study was cross-sectional in nature, as such, temporality cannot be ascertained in this study, and this association cannot be reported as a causal relationship. This is important to note since the same underlying social determinants of health that result in poor SRH outcomes (poverty, gender norms), can also impact school attendance and equitable access to education.

Despite the limitations, this study has notable strengths. For one, this study had a relatively large sample size and low degrees of missingness, contributing to high power. Additionally, the sampling approach allowed for the collection of data from a representative sample of adolescent girls living in urban and rural areas of Homa Bay and Narok counties. Lastly, to better understand the association of school attendance and the main SRH outcomes, we also presented descriptive statistics that aimed to provide contextual understanding of the study population and their SRH knowledge and behaviours.

## Conclusion

School attendance was independently associated with ever having sex, ever being pregnant, and condom use at last sex in this study. Out-of-school adolescent girls are highly vulnerable to adverse SRH outcomes and interventions are urgently needed to support keeping adolescent girls enrolled in and attending primary and secondary education. School represents an important avenue to disseminate SRH knowledge through CSE, which may in turn improve SRH outcomes for adolescents. Finally, adolescent girls need to be able to freely access and use modern contraceptives without fear of stigma and discrimination or financial burden, to ensure that adolescent girls have fewer unwanted and early pregnancies that disrupt their education. Given the integral role of school attendance in adolescent girls’ SRH outcomes, policy objectives that aim to financially support girls’ education is critical. In addition, our finding further underscores the lack of CSE among adolescent girls in Kenya which can further contribute to poor SRH outcomes among this population. As such the results highlight an urgent need for coordination among the government, community, and schools are required to develop and implement CSE.

## Data Availability

Data are available upon reasonable request.

## Abbreviations

SRH: Sexual and reproductive health
ITH: In their hands
STIs: Sexually transmitted infections
CSE: Comprehensive sexuality education
SSA: Sub-Saharan Africa
AOR: Adjusted odds ratio
SD: Standard deviation
CI: Confidence intervals
EMM: Effect measure modification
IUD: Intrauterine device
CT-OVC: Cash-transfer to orphaned and vulnerable children
UNESCO: United Nations Educational, Scientific and Cultural Organization
